# BiOCl Decorated NaNbO_3_ Nanocubes: A Novel p-n Heterojunction Photocatalyst With Improved Activity for Ofloxacin Degradation

**DOI:** 10.3389/fchem.2018.00393

**Published:** 2018-10-02

**Authors:** Jingjing Xu, Bingbing Feng, Ying Wang, Yadi Qi, Junfeng Niu, Mindong Chen

**Affiliations:** ^1^Jiangsu Key Laboratory of Atmospheric Environment Monitoring and Pollution Control, School of Environmental Science and Engineering, Collaborative Innovation Center of Atmospheric Environment and Equipment Technology, Jiangsu Engineering Technology Research Center of Environmental Cleaning Materials, Nanjing University of Information Science and Technology, Nanjing, China; ^2^Research Center for Eco-Environmental Engineering, Dongguan University of Technology, Dongguan, China

**Keywords:** photocatalysis, BiOCl, NaNbO_3_, p–n junction, ofloxacin

## Abstract

BiOCl/NaNbO_3_ p-n heterojunction photocatalysts with significantly improved photocatalytic performance were fabricated by a facile *in-situ* growth method. The obtained BiOCl/NaNbO_3_ samples were characterized by UV-vis absorption spectroscopy, scanning electron microscopy (SEM), X-ray diffraction (XRD), photocurrent (PC) and photoluminescence spectroscopy (PL). The photocatalytic activity of the BiOCl/NaNbO_3_ samples was investigated by the degradation of a typical antibiotic Ofloxacin (OFX). The experimental results showed that BiOCl/NaNbO_3_ composites exhibited much higher photocatalytic activity for OFX degradation compared to pure NaNbO_3_ and BiOCl. The degradation percent of OFX reached 90% within 60 min, and the apparent rate constant was about 8 times as that of pure NaNbO_3_ and BiOCl. The improved activity can be attributed to the formation of p-n junction between NaNbO_3_ and BiOCl. The formed p-n junction facilitated the separation of photogenerated holes and electrons, thereby enhancing photocatalytic activity. In addition, the composite photocatalyst showed satisfactory stability for the degradation of OFX. Due to the simple synthesis process, high photocatalytic activity, and the good recyclability of these composite photocatalysts, the results of this study would provide a good example for the rational design of other highly efficient heterojunction photocatalytic materials.

## Introduction

The problem of water pollution has attracted increasing attention worldwide due to many toxic pollutants continue to enter into our water bodies (Ao et al., 2016b; Li et al., 2018; Yuan et al., 2018). As a green and sustainable technology, semiconductor photocatalysis can completely eliminate most pollutants in water and air (Zhang et al., 2016, 2017a,b; Chen et al., 2018; Guo et al., 2018; He et al., 2018; Yang et al., 2018). The contaminants are degraded by hydroxyl radical or other free radicals which are produced in the process after the absorption of light (Li et al., 2015; Cao et al., 2016; Xu et al., 2016; Yu et al., 2016; Zhang and Xiao, 2017; Han et al., 2018; Zhong et al., 2018). There are many semiconductors (such as oxides, sulfides, nitrates and silver based compounds etc.) can be served as photocatalysts to induce the degradation of pollutants.

In recent years, NaNbO_3_ has attracted much attention due to its unique properties of nonlinear optics, ferroelectric, ionic conduction, photorefractive and photocatalytic properties (Katsumata et al., 2010; Lv et al., 2010; Li et al., 2012a, 2013; Fan et al., 2015; Qian et al., 2018). Because NaNbO_3_ is a typical n type semiconductor with unique crystal [NbO6] angle eight-hedral crystal structure, it has the advantage of relative high transfer efficiency of electrons during the photocatalytic process (Shi et al., 2014b; Song et al., 2015). Recent studies have shown that NaNbO_3_ can be used as an efficient photocatalyst for the evolution of H_2_ and the reduction of CO_2_ (Li et al., 2012b, 2014). In addition, NaNbO_3_ also shows the ability for the decomposition of organic pollutants (Li et al., 2008; Li, 2010; Chen et al., 2014). However, because NaNbO_3_ has a large band-gap, it cannot be used as a visible light responsive photocatalyst. Various experiments have shown that forming heterojunction with other semiconductor materials was a promising way to improve the photocatalytic activity of NaNbO_3_. For example, an effective composite photocatalyst NaNbO_3_/WO_3_ has been proved to has stronger dye degradation activity compared with the corresponding single component (Shifu et al., 2013). Moreover, other NaNbO_3_ based composite photocatalysts, such as AgO_2_/NaNbO_3_ (Zhang et al., 2017), Pt/NaNbO_3_ (Liu et al., 2016) and CdS/NaNbO_3_ (Kumar et al., 2014), were also successfully prepared for photocatalytic degradation of pollutants.

On the other hand, although BiOCl (a typical p-type oxyhalide semiconductor) has been extensively investigated in the field of photocatalysis (Ye et al., 2014; Ao et al., 2016a; Hu et al., 2016; Mao et al., 2016; Ma Y. C. et al., 2016; Wang et al., 2018). However, the recombination rate of photogenerated charges in pure phase of BiOCl is high, resulting in its limited photocatalytic activity under UV light. It has recently been found that the photocatalytic activity of BiOCl can be promoted through the formation of p-n junctions with n type semiconductors (Rajeshwar et al., 2001; Wang et al., 2012; Fang et al., 2016). The enhanced activity can be ascribed to the fact that the internal electric field at the p-n junction interface improved the separation and transfer of photo-generated charges (Yan et al., 2017). However, as far as we know, the preparation and activity of BiOCl/NaNbO_3_ p-n heterojunction photocatalyst have not been studied yet.

In the present work, we constructed a new type of p-n heterojunction photocatalyst (BiOCl/NaNbO_3_) by a simple *in situ* growth method. Compared with pure BiOCl and NaNbO_3_, the composite has better photocatalytic activity for the degradation of ofloxacin (OFX). The experimental results proved that the p-n junctions formed between BiOCl/NaNbO_3_ composites promote the transfer and separation of photogenerated electron-hole pairs, thus enhancing the photocatalytic activity.

## Experimental

### Synthesis of NaNbO_3_ cubes

All the reagents and materials used in this experiment were analytical grades. All of them were purchased from China pharmaceutical chemical reagents Co., Ltd. NaNbO_3_ was directly synthesized by hydrothermal method described as following. 2 g Nb_2_O_5_ was added to the 120 mL NaOH solution (10 M) and stirred for 120 min before the suspension was transferred to the 200 ml Teflon-lined stainless steel autoclave. The reaction kettle was kept at 150°C for 48 h. Subsequently, the autoclave was cooled to room temperature naturally after the reaction. Then remove the reactor and precipitate it naturally, pour the supernatant out of the precipitate and centrifugate, then wash it with deionized water and ethanol for 3–5 times. Finally, the product was dried for 12 h in a vacuum oven at 60°C.

### Synthesis of BiOCl/NaNbO_3_ p–n junction photocatalysts

BiOCl/NaNbO_3_ heterostructures were prepared by a simple *in-situ* growth method. A certain amount of Bi(NO_3_)_3_^.^5H_2_O was added to 300 mL of ultrapure water and stirred for 1 h to form solution A. Meanwhile, NaNbO_3_ powder was dispersed in 80 mL ultrapure water and sonicated for 30 min to form solution B. A certain amount of KCl was added to 60 mL ultrapure water to form solution C. Finally, B and C were slowly added to A under rapid stirring. Afterwards, the stirring was continued for 24 h. The product was then filtered and thoroughly washed with distilled water and ethanol before it was dried at 60°C for 24 h. By Changing the amount of Bi(NO_3_)_3_·5H_2_O and KCl added, NaNbO_3_/BiOCl composites with different theoretical BiOCl mass ratios of 10, 25, 75, and 100% (expressed as BN-1, BN-2, BN-3, and BN-4) were produced. In order to facilitate comparison, pure BIOCl samples are also prepared in the same way, that is to say, no NaNbO_3_ is added in the process of making composite samples.

### Characterization of photocatalysts

The results of X ray diffraction (XRD) scanning of pure BiOCl, NaNbO_3_ and BiOCl/NaNbO_3_ were recorded by Shimadzu XRD 6100 X-ray diffractometer. The morphology and microstructure of the samples were characterized by scanning electron microscopy (SEM, Hitach S-4800). The band gap (Eg) of the sample was measured by UV-vis absorption spectroscopy at 200~800 nm at room temperature, and the integrated ball attachment was installed on the ultraviolet visible spectrophotometer (UV-3600). Photocurrent (PC) and mott-Schottky (MS) were carried out on the traditional three electrode system (the electrochemical workstation of CHI-660D Chenhua chemical equipment company in Shanghai, China). The contrast electrode and the reference electrode are platinum electrodes and Ag/AgCl electrodes respectively.

### Photocatalytic activity experiments

The experimental vessel is a double-layer quartz beaker with cooling water circulation system for photocatalytic reaction. During the experiment, the temperature of the solution can be maintained at about 25 °C, so as to reduce the influence of temperature on the experiment. The light source used in the photocatalytic process is a 300 W xenon lamp(200 nm < λ < 400 nm), and before irradiation, 25 mg photocatalyst was added to 50 mL OFX solution (5 mg/L). The suspension was stirred magnetically in the dark for 30 min to achieve adsorption/desorption equilibrium between photocatalysts and OFX. During the photocatalytic process, a certain suspension was sampled at the prescribed time intervals. The sampled suspension was centrifuged before the determination of OFX concentration.

## Results and discussion

### Characterizations

**Figure 1:**
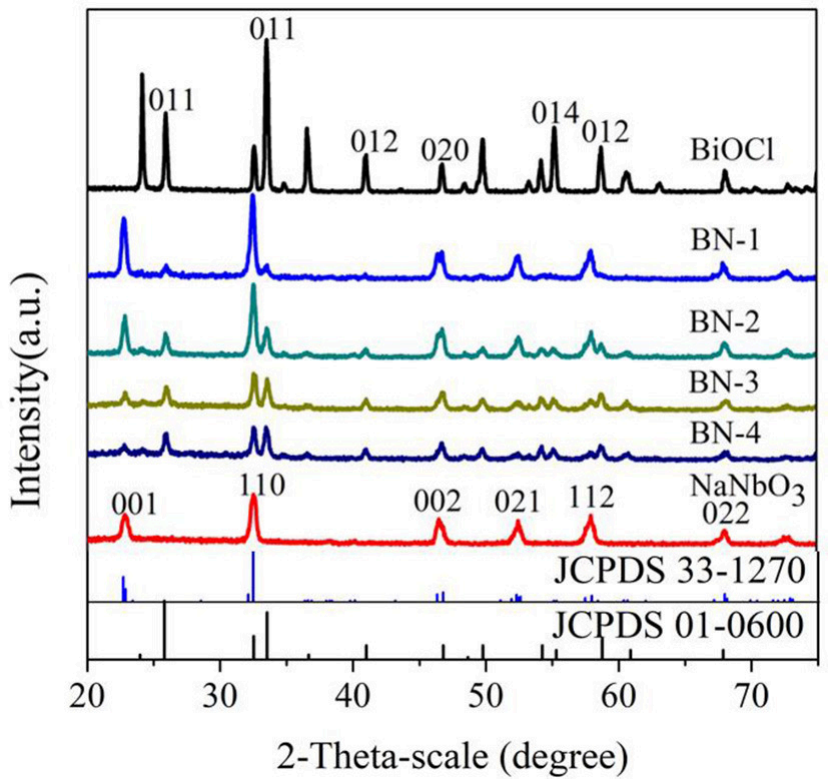
XRD patterns of BiOCl, NaNbO_3_, and BiOCl/NaNbO_3_ composites with different BiOCl contents.

The XRD diagrams of pure BiOCl, NaNbO_3_ and BiOCl/NaNbO_3_ composites were shown in Figure 1. It can be seen that there are seven different diffraction peaks, which can be indexed to tetragonal BiOCl (JCPDS card number 01-0600) (Ma W. et al., 2016). Sharp and narrow diffraction peaks indicate good crystallinity for pure BiOCl. From the pattern of pure NaNbO_3_, several main diffraction peaks can be seen at 22.7, 32.5, 46.4, 52.7, 58.1, and 68.11° corresponding to (001), (110), (002), (021), (112), and (022) planes of NaNbO_3_ orthorhombic phases (JCPDS card number 33-1270) (Saito and Kudo, 2013). The diffraction peaks of pure BiOCl and NaNbO_3_ and the diffraction peaks of heterostructure BiOCl/NaNbO_3_ composites can be clearly observed by XRD diagram. In addition, when the mass ratio of BiOCl to NaNbO_3_ increases from 10 to 100%, the diffraction peak intensity of BiOCl increases and the corresponding NaNbO_3_ peak intensity decreases. Furthermore, when BiOCl grows on NaNbO_3_, its diffraction peak is wider than that of pure BiOCl. This phenomenon indicates that BiOCl on the surface of NaNbO_3_ cube has smaller particle size. The result indicates that the addition of NaNbO_3_ can effectively inhibit the growth of BiOCl (Shang et al., 2009). XRD showed that the samples were clean and had no other phase of impurity, so the composites were basically composed of BiOCl and NaNbO_3_.

**Figure 2:**
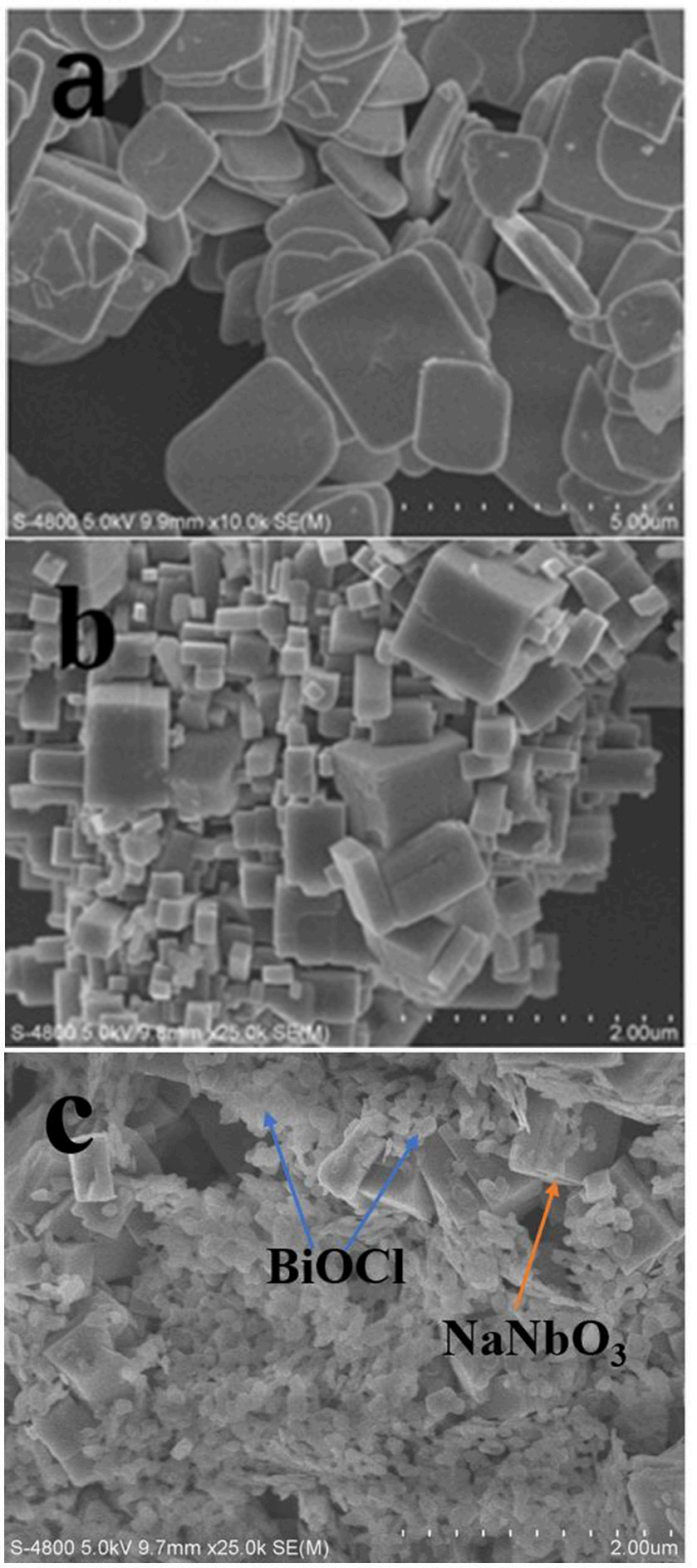
SEM images of pure BiOCl **(a)**, NaNbO3 **(b)** and BN-3 **(c)**.

The morphology and structure of BiOCl, NaNbO_3_, and BiOCl/NaNbO_3_ composites can be clearly seen by SEM characterization. The SEM image of pure BiOCl is displayed in Figure 2a. As can be seen from the graph, pure BiOCl is composed of regular, square like nano plates. At the same time, we can see that the synthesized NaNbO_3_ has irregular cubic shape and smooth surface in Figure 2b. A typical SEM image of the BN-3 composite is shown in Figure 2c. It can be seen that NaNbO_3_ cubes are anchored by BiOCl nanosheets which are thiner and smaller than pure BiOCl. During the in-sith growth process, the NaNbO_3_ particles played a role of heterogeneity and inhibited the growth of BiOCl to lager ones. After analyzing the SEM image, the conclusion is in good agreement with the previous XRD analysis. BiOCl and NaNbO_3_ form p-n junctions at the interface because of the close contact between BiOCl and NaNbO_3_. This improves the carrier transport rate and separation efficiency in photocatalysis and helps to improve the activity.

**Figure 3:**
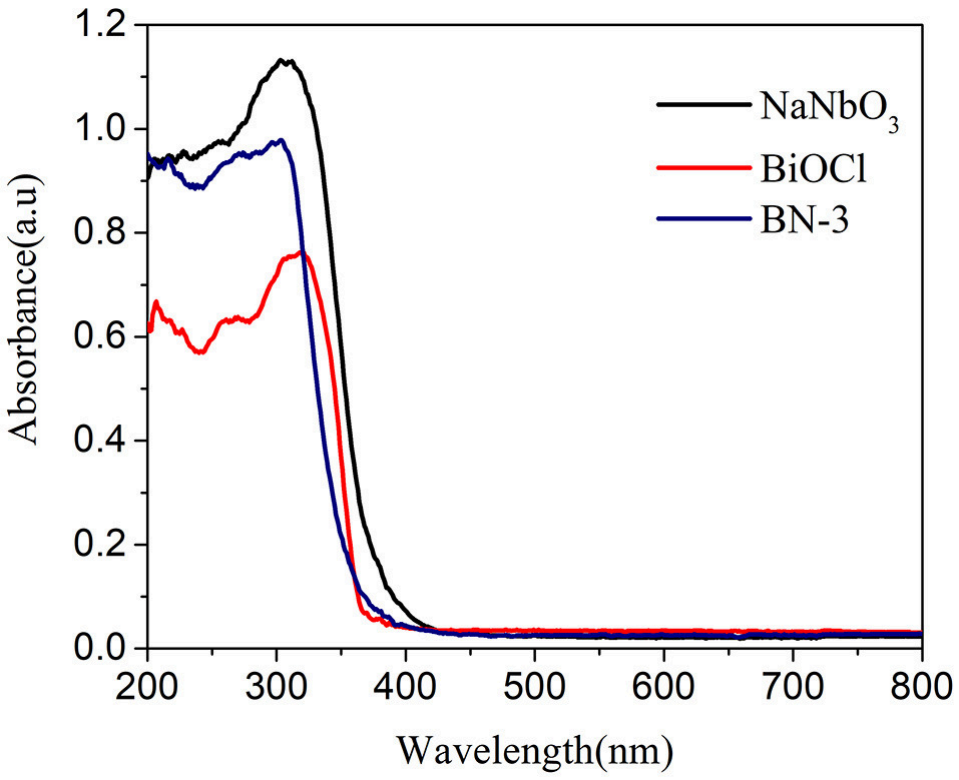
UV-vis absorption spectra of BiOCl, NaNbO_3_ and BN-3.

The optical properties of the samples have a great influence on the utilization of sunlight. Therefore, UV-DRS for pure BiOCl, NaNbO_3_ and BiOCl/NaNbO_3_ composites are investigated. As we can see from Figure 3, the absorbance threshold of NaNbO_3_ and pure BiOCl are at about 400 and 380 nm, respectively. The band-gap of photocatalysts can be obtained from the following equation:

$$\begin{array}{l} \text{Eg}=1240/\lambda , \end{array}$$

where Eg is the band-gap energy and λ is the cut off wavelength. Therefore, the band gaps of NaNbO_3_ and BiOCl prepared were 3.10 and 3.26 eV, respectively, which are similar to those reported previously (Shi et al., 2014a; Ma W. et al., 2016). It can also be seen from Figure 3 that BiOCl/NaNbO_3_ exhibits higher UV absoption performance compared to pure BiOCl. Furthermore, the band edge of BiOCl/NaNbO_3_ exhibits a little red shift compared to pure BiOCl.

**Figure 4:**
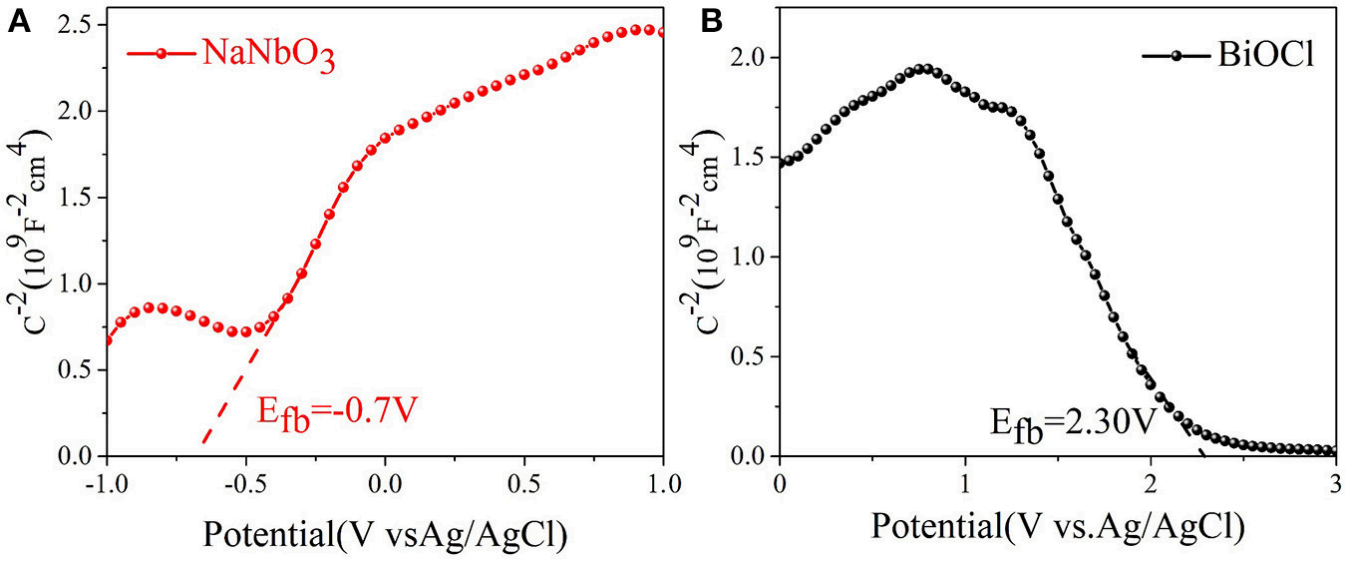
The Mott-Schottky plots of **(A)** NaNbO_3_ and **(B)** BiOCl.

Through the band structure of the catalyst, we further explore the way of carrier transfer. The type and surface potential of NaNbO_3_ and BiOCl semiconductors are determined by the Mott-Schottky plots. The positive tangent in the graph indicates that the sample is n type semiconductor, while the negative tangent in the graph indicates that the sample is p type semiconductor (Kuang et al., 2015). The results shown in Figures 4A,B show that BiOCl and NaNbO_3_ are p-type and n-type semiconductors, respectively. In addition, the flat potential (relative to Ag/AgCl) of BiOCl and NaNbO_3_ are 2.30 V and −0.70 V respectively through the intersection point between tangent and abscissa. According to the following equation, the potential (relative to Ag/AgCl) can be converted to a normal hydrogen electrode (NHE). (1) (Zhou et al., 2017; Liu et al., 2018):

$$\begin{array}{l} {\text{E}}_{\text{NHE}}={\text{E}}_{\text{Ag}/\text{AgCl}}+{\text{E}}_{\text{Ag}/\text{AgCl}}^{0} \end{array}$$

where ${\text{E}}_{\text{Ag}/\text{AgCl}}^{0}$ = 0.197 V. In particular, the maximum of the valence band (VB) is about 0.1 V lower than the flat potential of the p-type semiconductor; the minimum value of the conduction band (CB) is about 0.1 V higher than the flat potential of the n-type semiconductor (Liu et al., 2017b). Therefore, the VB position of BiOCl and the CB position of NaNbO_3_ are 2.60 and −0.60 V (vs. NHE), respectively. The Eg of BiOCl and NaNbO_3_ were 3.26 and 3.10, respectively. Therefore, according to Eg. (2) (Liu et al., 2017a):

$$\begin{array}{l} \text{Eg}={\text{E}}_{\text{VB}}-{\text{E}}_{\text{CB}} \end{array}$$

the position of CB and VB for BiOCl and NaNbO_3_ are −0.66 and 2.50 V, respectively.

### Photocatalytic activity

**Figure 5:**
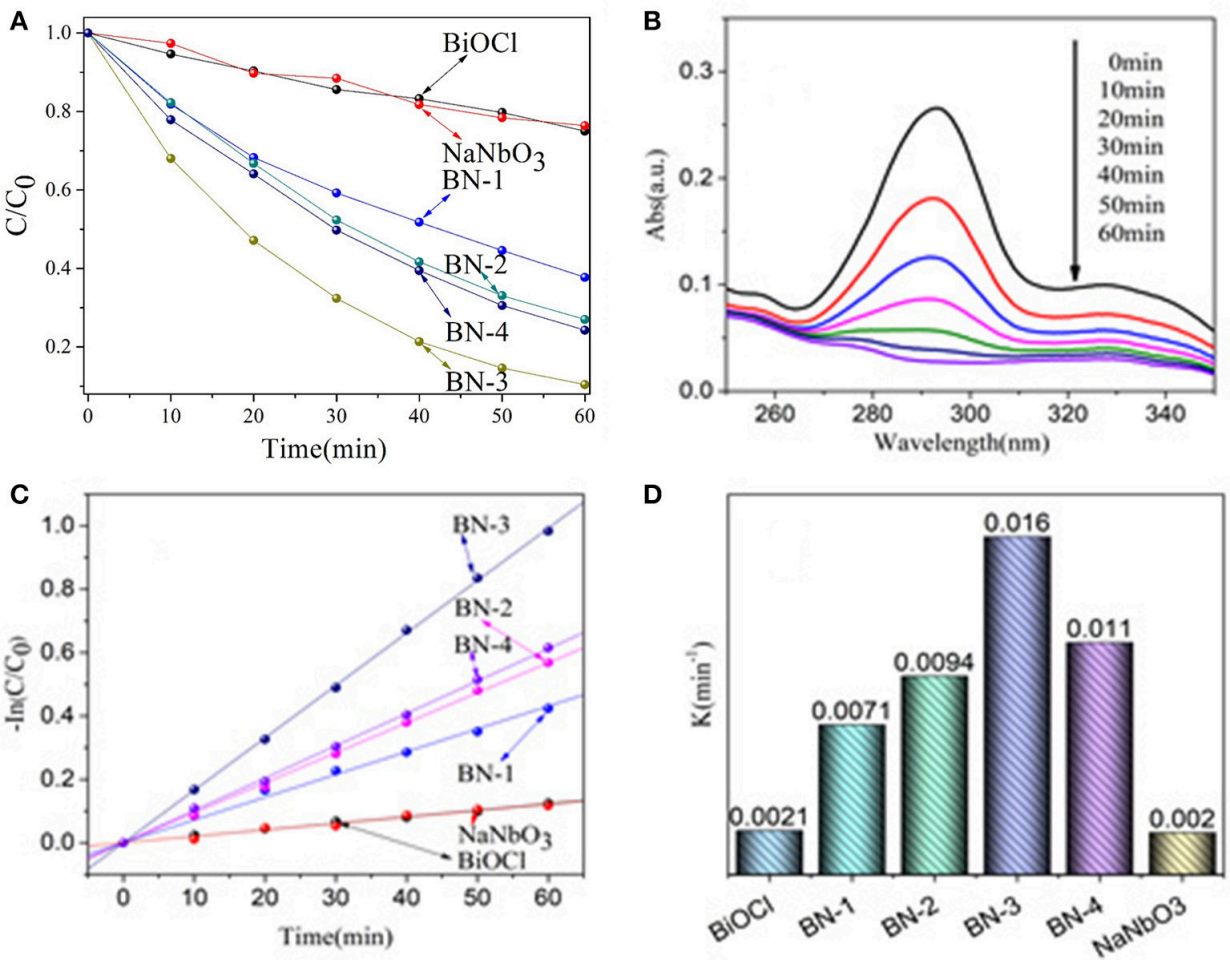
**(A)** Photocatalytic activities of NaNbO_3_, BiOCl and BiOCl/NaNbO_3_ composites for the degradation of OFX (5 mg/L) under UV light irradiation (200 nm < λ < 400 nm), **(B)** UV spectral changes of OFX in aqueous BN-3 composites dispersions as a function of irradiation time, **(C)** The kinetics of OFX degradation, and **(D)** The degradation rateconstants.

The photocatalytic activity of BiOCl/NaNbO_3_ composites with p-n junctions is mainly studied on the degradation of OFX under UV irradiation. As shown in Figure 5A, In the presence of NaNbO_3_ or BiOCl, about 20% OFX are decomposed within 60 min. It can also be seen that all BiOCl/NaNbO_3_ composites exhibit significantly higher photocatalytic activity than that of pure BiOCl and NaNbO_3_. In addition, it was found that the mass ratio of BiOCl has a significant effect on the activity of BiOCl/NaNbO_3_ composites. Specifically, the activity increase gradually when the BiOCl content is increased from 10 to 75%. When the content of BiOCl is further increased to 100%, the activity of the sample begin to decrease. Therefore, sample BN-3 with BiOCl mass ratio of 75% exhibites the highest activity (the degradation percent of OFX is about 90% after 60 min irradiation). The time evolution of the OFX solution spectra in the degradation process by BN-3 were measured and are shown in Figure 5B. The absorption peak of OFX decreases as the increase of irradiation time and reaches the minimum at 60 min.

It has also been found that the photocatalytic degradation of OFX follows pseudo-first order kinetic calculated from the following formula (as shown in Figure 5C):

$$\begin{array}{l} -\text{ln}\left({\text{C}}_{\text{t}}/{\text{C}}_{0}\right)=\text{kt} \end{array}$$

The obtained k of OFX removal rate of pure BiOCl, NaNbO_3_ and BiOCl /NaNbO_3_ composites can be calculated by this formula. In Figure 5D, it is more clear to see that the kinetic constants of BiOCl, NaNbO_3_, BN-1, BN-2, BN-3, and BN-4 are 0.0021, 0.002, 0.0071, 0.0094, 0.016, and 0.011 min^−1^, respectively. Therefore, compared with the single phase BiOCl and NaNbO_3_, the BiOCl/NaNbO_3_ composite can promote the separation of the electron-hole pairs on the interface, thus promoting the enhancement of the photocatalytic activity.

**Figure 6:**
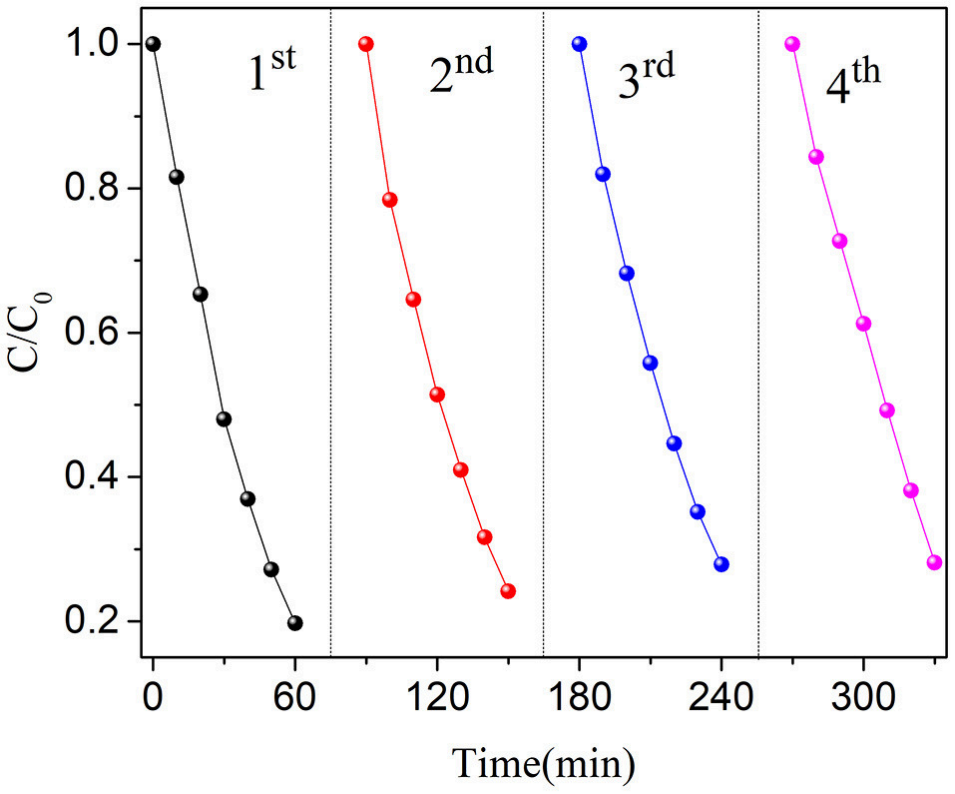
Cyclic operation of BN-3 under UV light.

The stability of one photocatalyst is very important to the practical application. Therefore, the recycle experiments are carried out for BN-3. As shown in Figure 6, the photocatalytic performance of the catalyst has only slight loss after four cycles of reutilization of the photocatalyst. This experimental phenomenon indicates that the photocatalyst is stable to the photodegradation of OFX. The experimental results show that the photocatalytic degradation of organic pollutants by the prepared BiOCl/NaNbO_3_ composites is stable, and it is of great significance for the practical application of the catalyst.

### Photocatalytic mechanism

**Figure 7:**
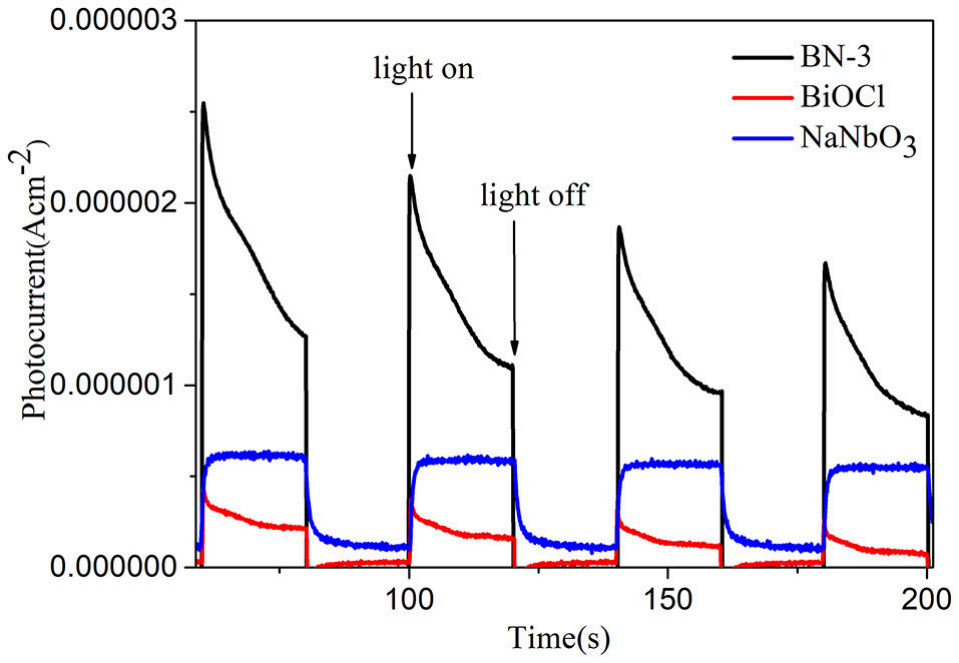
Transient photocurrent responses for NaNbO_3_, BiOCl, and BN-3 composite.

**Figure 8:**
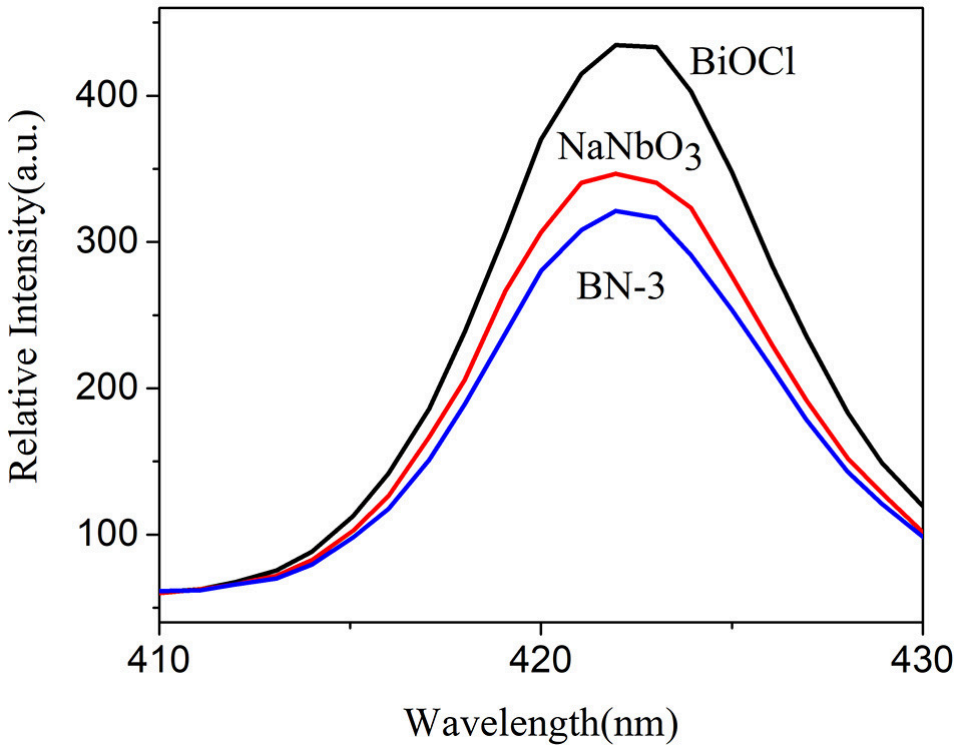
PL emission spectra of NaNbO_3_, BiOCl, and BN-3 composite.

The interfacial charge transfer kinetics between BiOCl and NaNbO_3_ can be further elucidated by photocurrent measurements. Figure 7 shows the photocurrent-time curves of the BiOCl, NaNbO_3_, and BN-3 composites under an on/off cycle of intermittent UV irradiation. As shown in Figure 7, BN-3 composite exhibits much higher photocurrent than that of pure BiOCl and NaNbO_3_. Therefore, the BN-3 can transfer and separate photogenerated electron hole pairs more efficiently under ultraviolet light irradiation. In addition, the photoluminescence (PL) spectra of pure BiOCl, NaNbO_3_ and BiOCl/NaNbO_3_ composite samples were tested to determine the separation efficiency of photo-generated charges. The excitation wavelength used in the test is 285 nm. In Figure 8, it is found that pure NaNbO_3_ is the strongest relative to other sample emission peaks within the range of 350–550 nm. For pure BiOCl samples, their emission peaks are relatively weak relative to pure NaNbO_3_. The PL luminescence strength of BN-3 composites is the lowest in the three samples. The results show that the combination of pure BiOCl and NaNbO_3_ can effectively inhibit the recombination of electron hole pairs. The results are in good agreement with the measurement results of photocurrent.

**Figure 9:**
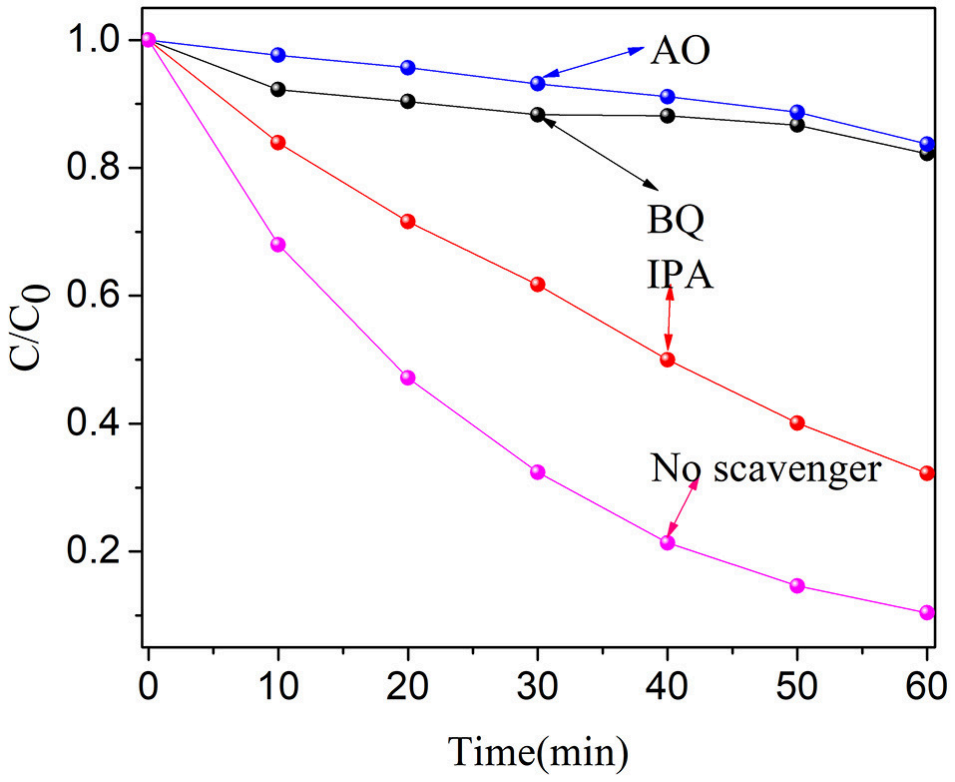
Trapping experiment of active species during the photocatalytic degradation of OFX over BN-3 under UV light irradiation.

In order to study the degradation mechanism, the main active species produced in the degradation process were determined. Therefore, three radical scavengers such as isopropanol (IPA, •OH scavenger) (Cao et al., 2012), ammonium oxalate (AO, h^+^ scavenger) (Chang et al., 2013) and p-benzoquinone (BQ, •${\text{O}}_{2^{-}}$scavenger) (Sun et al., 2017) have been added separately in the degradation systems. When BQ and AO were introduced, the degradation rate of OFX decreased significantly (see Figure 9). Moreover, the degradation rate of OFX was also decreased in the presence of IPA. These results indicate that h^+^ and •${\text{O}}_{2^{-}}$ play an important role in the degradation process. •OH has a certain effect on the degradation of OFX. Therefore, we can conclude that the free radicals of •${\text{O}}_{2^{-}}$, h^+^ and •OH in active species play a certain role in the degradation of OFX.

**Scheme 1:**
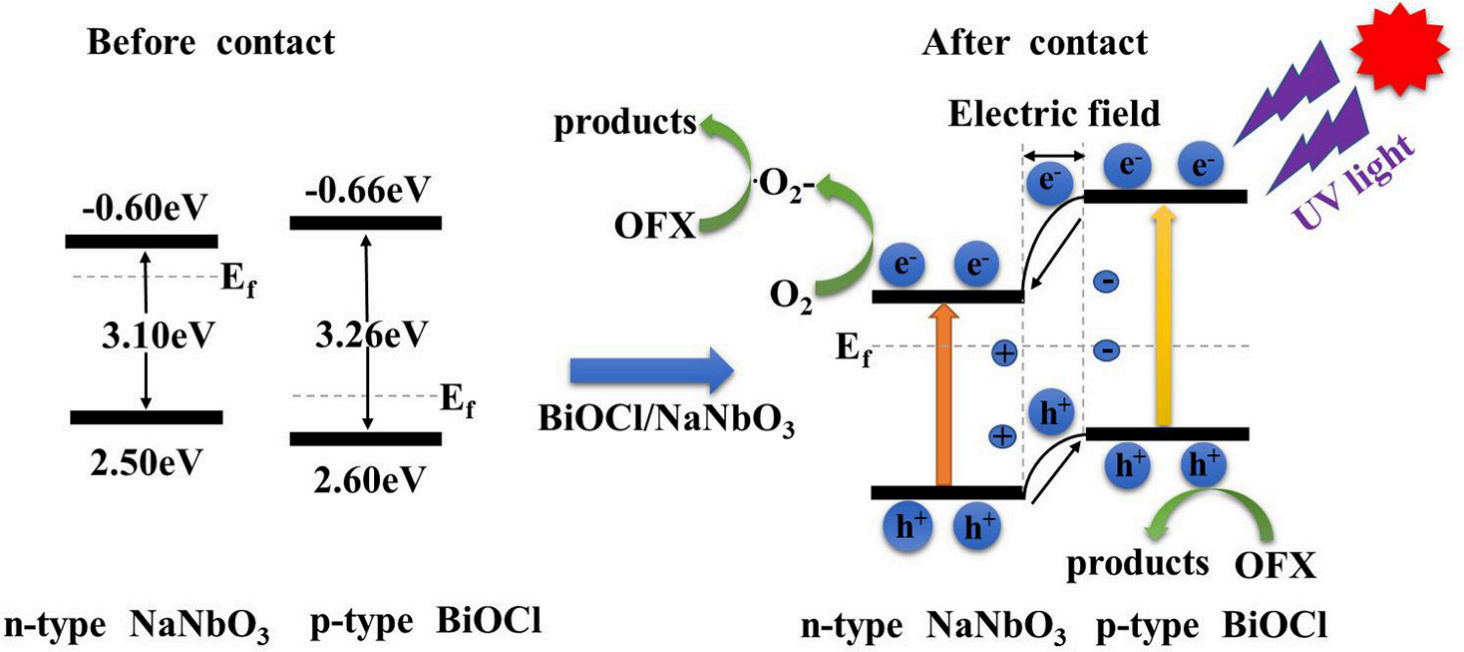
Proposed possible mechanism of the photocatalytic degradation of pollutants over BiOCl/NaNbO_3_ p–n junction under UV light irradiation.

The formation of heterojunction in the composite system has a positive effect on the separation of photogenerated electrons and holes. Based on the above experimental results, we propose a reasonable photocatalytic mechanism for BiOCl/NaNbO_3_ (Scheme 1). The enhanced photocatalytic activity of BiOCl/NaNbO_3_ heterojunction originates from the unique matching band location of these two semiconductors after forming heterojunction. Based on the experimental data, we know that the band gap of BiOCl is larger than that of NaNbO_3_. The Fermi level (E_f_) of BiOCl is located near the valence band because it is a p type semiconductor, and the Fermi level of n type semiconductor NaNbO_3_ approaches the conduction band. When the mixture of BiOCl and NaNbO_3_ forms a p-n type heterojunction of BiOCl/NaNbO_3_, the Fermi level of BiOCl moves upwards, while the Fermi level of the NaNbO_3_ moves downward until the Fermi level of BiOCl and NaNbO_3_ is at the same level. Finally, the CB position of BiOCl becomes higher than NaNbO_3_ after moving up. At the same time, BiOCl and NaNbO_3_ form an internal electric field in the final equilibrium state. At the same time, the valence band (VB) of NaNbO_3_ is also lower than that of BiOCl. Due to the special band structure of the BiOCl/NaNbO_3_ composite, the photoelectrons are excited from the VB of the BiOCl to the CB, because the special ladder like structure of the BiOCl/NaNbO_3_ heterostructure leads to the electronic transfer from CB of BiOCl to the CB of NaNbO_3_. Electrons stored in NaNbO_3_'s CB can react with dissolved O_2_ to form •${\text{O}}_{2^{-}}$, while the adsorbed hydroxy or H_2_O molucules can also react with h^+^ to form •OH or directly oxidize organic pollutants. Therefore, the formed p-n junction between BiOCl and NaNbO_3_ provide an effective electron transfer path to reduce the recombination of electron-hole pairs, thus improving the activity of the as fabricated BiOCl/NaNbO_3_ composites.

## Conclusions

In this work, BiOCl/NaNbO_3_ composites with p-n heterojunctions were synthesized by simple hydrothermal method. Through experiments and characterization, it is confirmed that p-n junctions formed on the interface between BiOCl and NaNbO_3_. The results indicted that the separation efficiency of photo-generated carriers has been greatly improved because of the formation of p-n junctions. Recycle experiments showed that the composite photocatalyst exhibited good stability. The experimental results also show that pollutants are mainly degraded by h^+^ and ·${\text{O}}_{2^{-}}$ radicals. Therefore, BiOCl/NaNbO_3_ heterojunction nanocomposites can be used as a highly efficient and reusable photocatalyst, which has potential applications in the removal of organic pollutants.

## Author contributions

JX and MC designed the project, guided the study and polished the manuscript. BF, YW, and YQ conducted the experiments and characterized the samples. JN revised the manuscript.

### Conflict of interest statement

The authors declare that the research was conducted in the absence of any commercial or financial relationships that could be construed as a potential conflict of interest.
